# Supplementary figures and images for: ABCB1 Amplicon Contains Cyclic AMP Response Element-Driven TRIP6 Gene in Taxane-Resistant MCF-7 Breast Cancer Sublines
Source: Genes (Basel). 2023 Jan 23;14(2):296. doi: 10.3390/genes14020296 (PMC9957548; doi:10.3390/genes14020296)

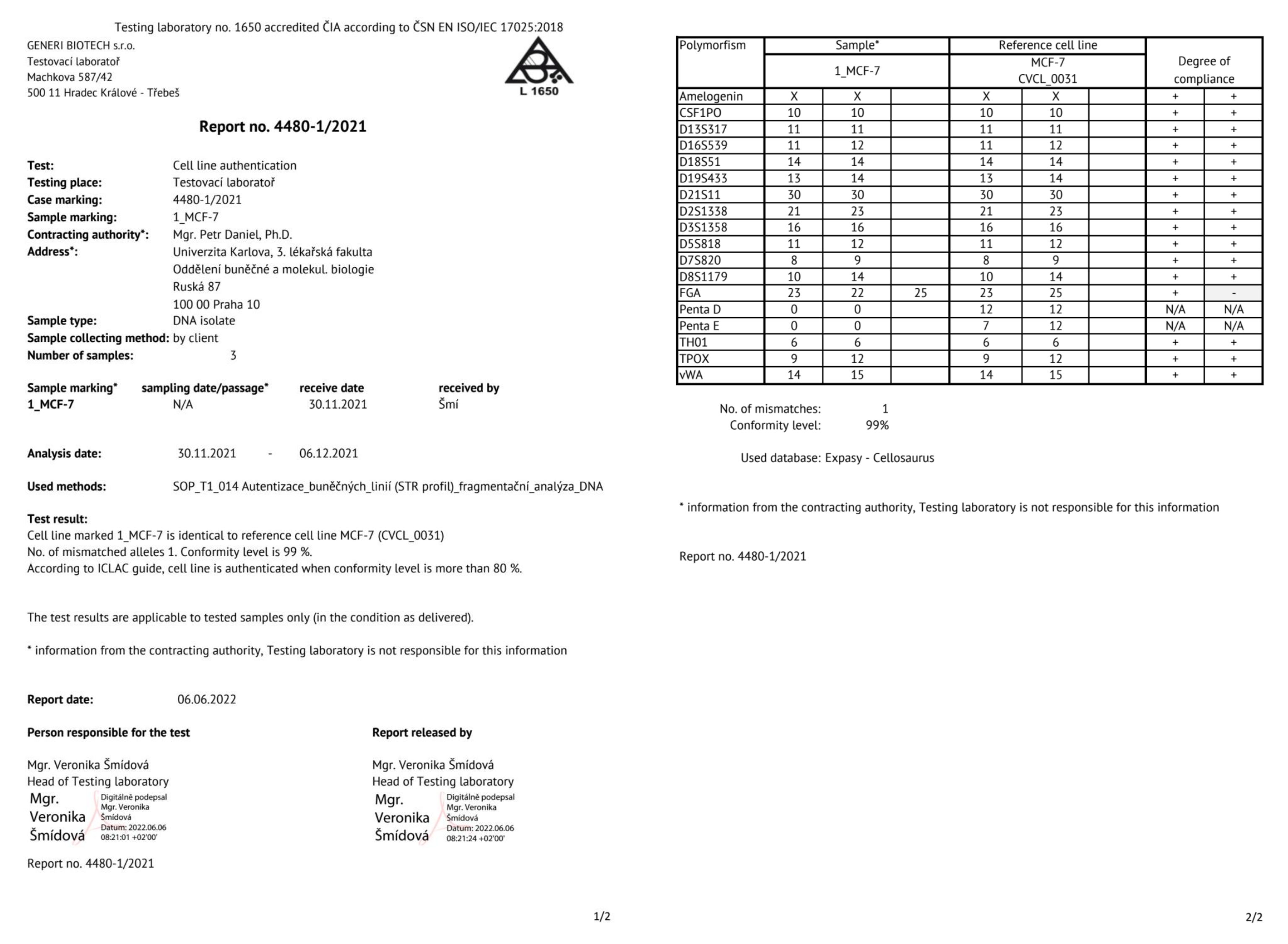

Supplement: Supplementary file 1 [file genes-14-00296-s001.zip › Supplementary Figure S1_Authentication report MCF-7.tif]

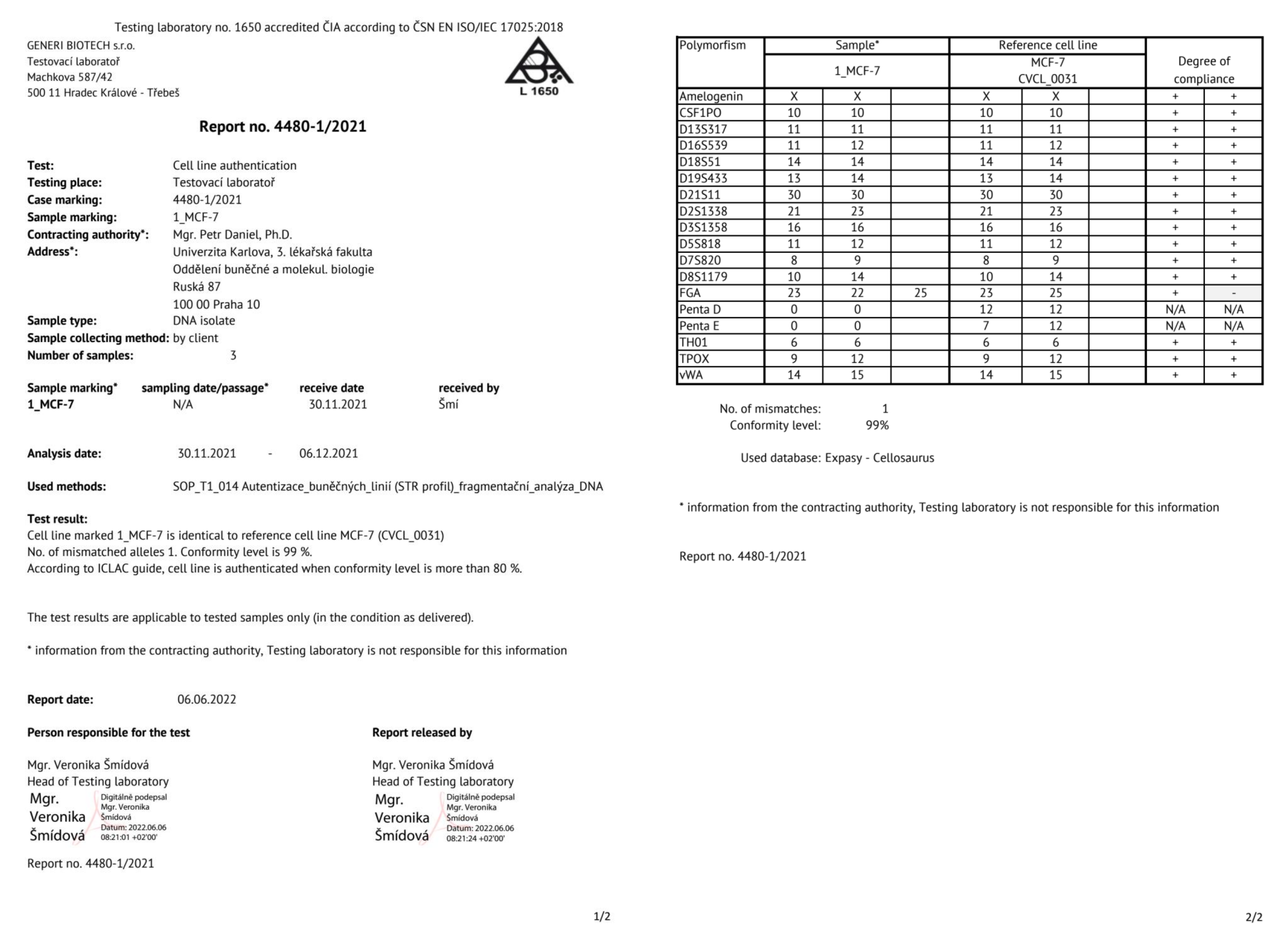

Supplement: Supplementary file 1 [file genes-14-00296-s001.zip › Supplementary Figure S2_Authentication report MCF-7 PacR.tif]

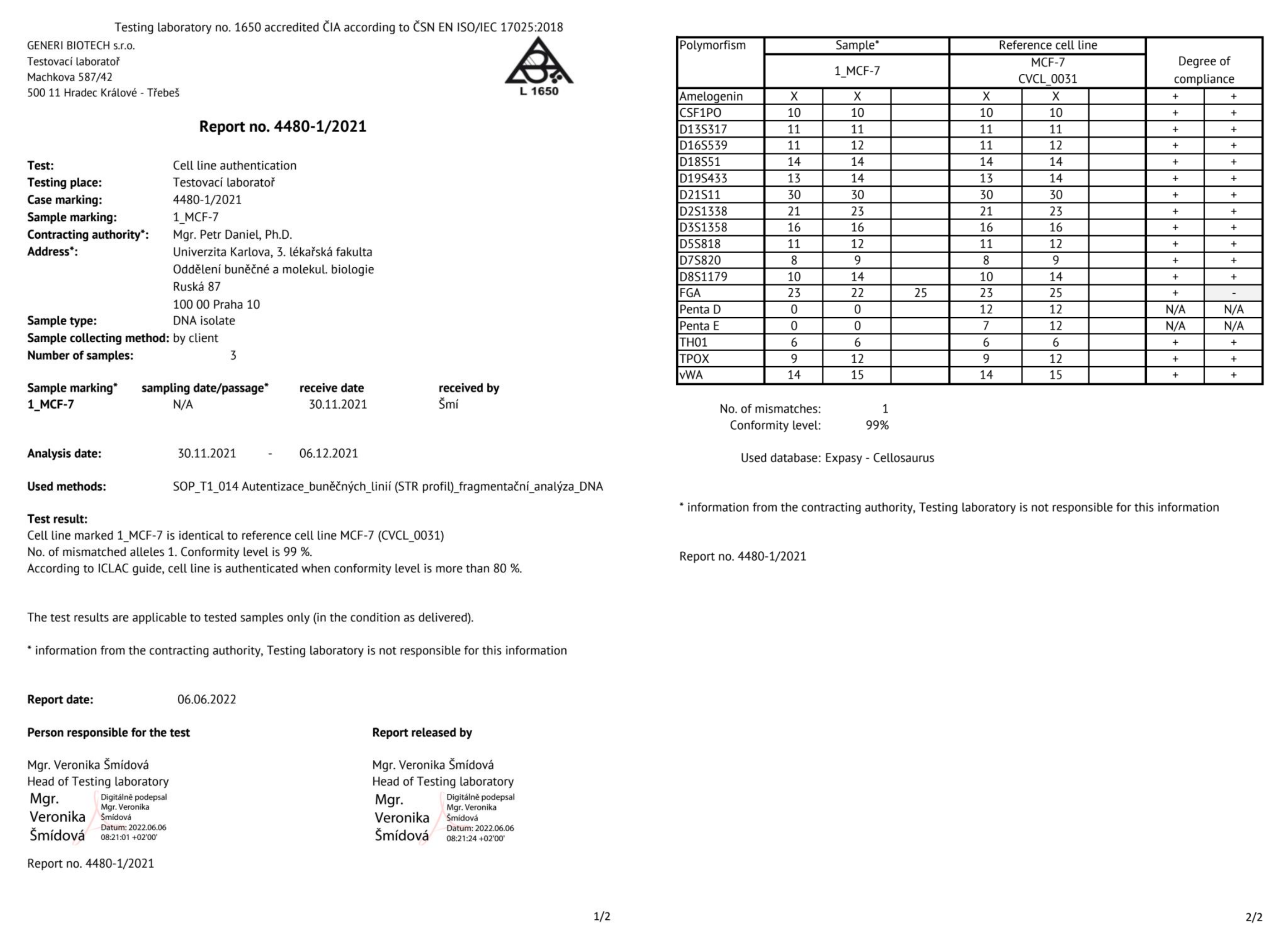

Supplement: Supplementary file 1 [file genes-14-00296-s001.zip › Supplementary Figure S3_Authentication report MCF-7 0035R.tif]

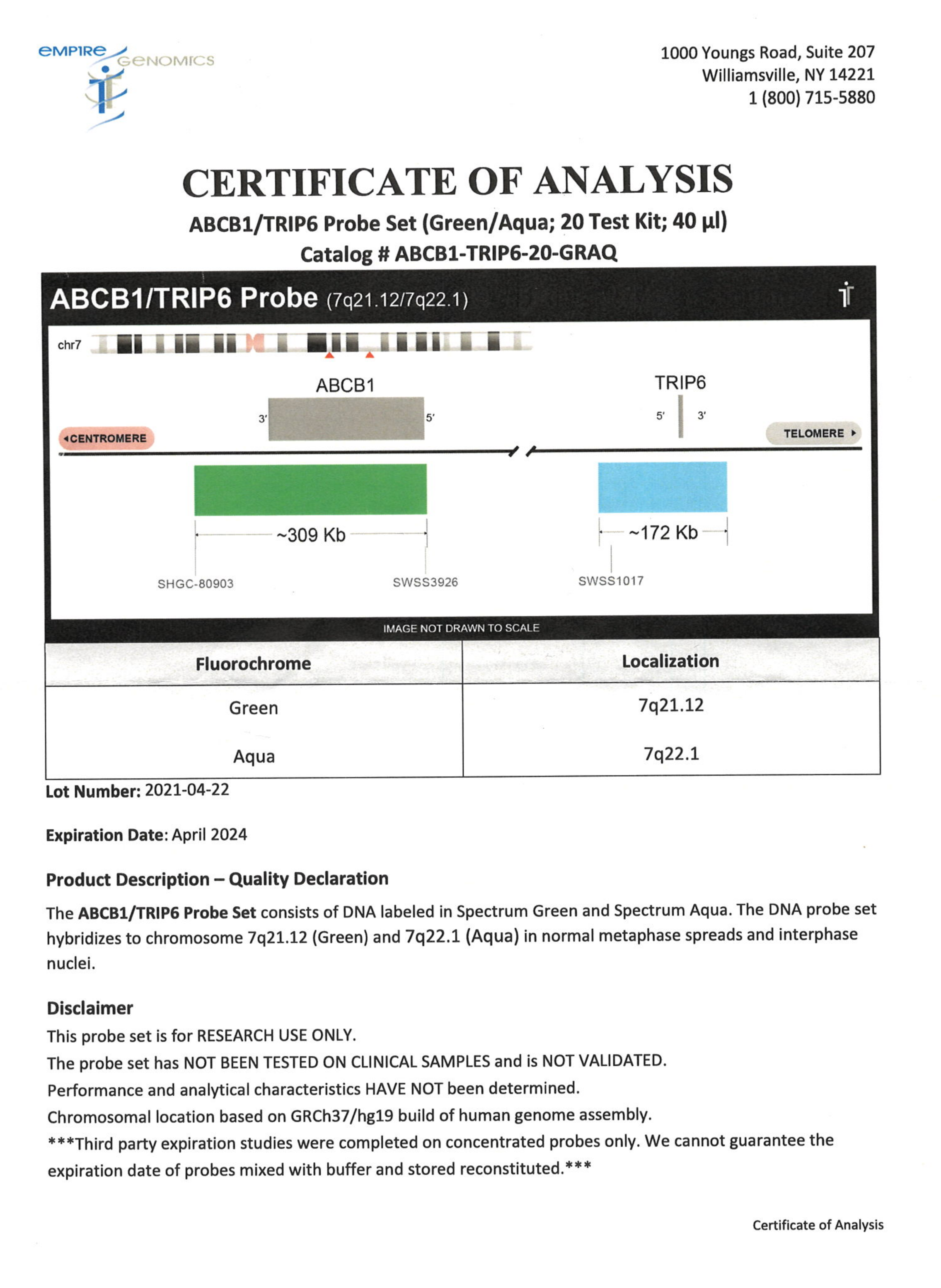

Supplement: Supplementary file 1 [file genes-14-00296-s001.zip › Supplementary Figure S4_Empire Genomics Certificate.tif]

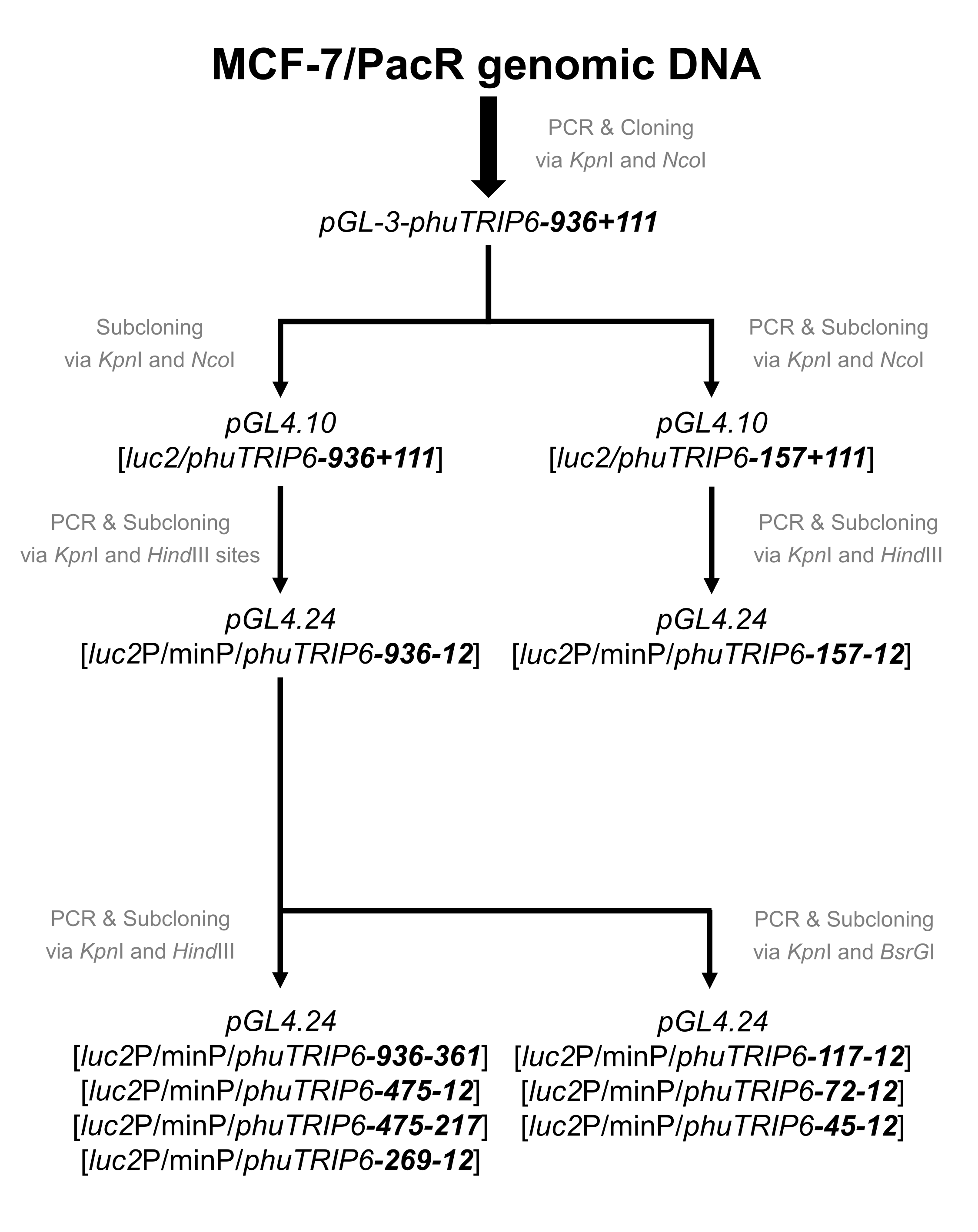

Supplement: Supplementary file 1 [file genes-14-00296-s001.zip › Supplementary Figure S5_Cloning scheme.tif]

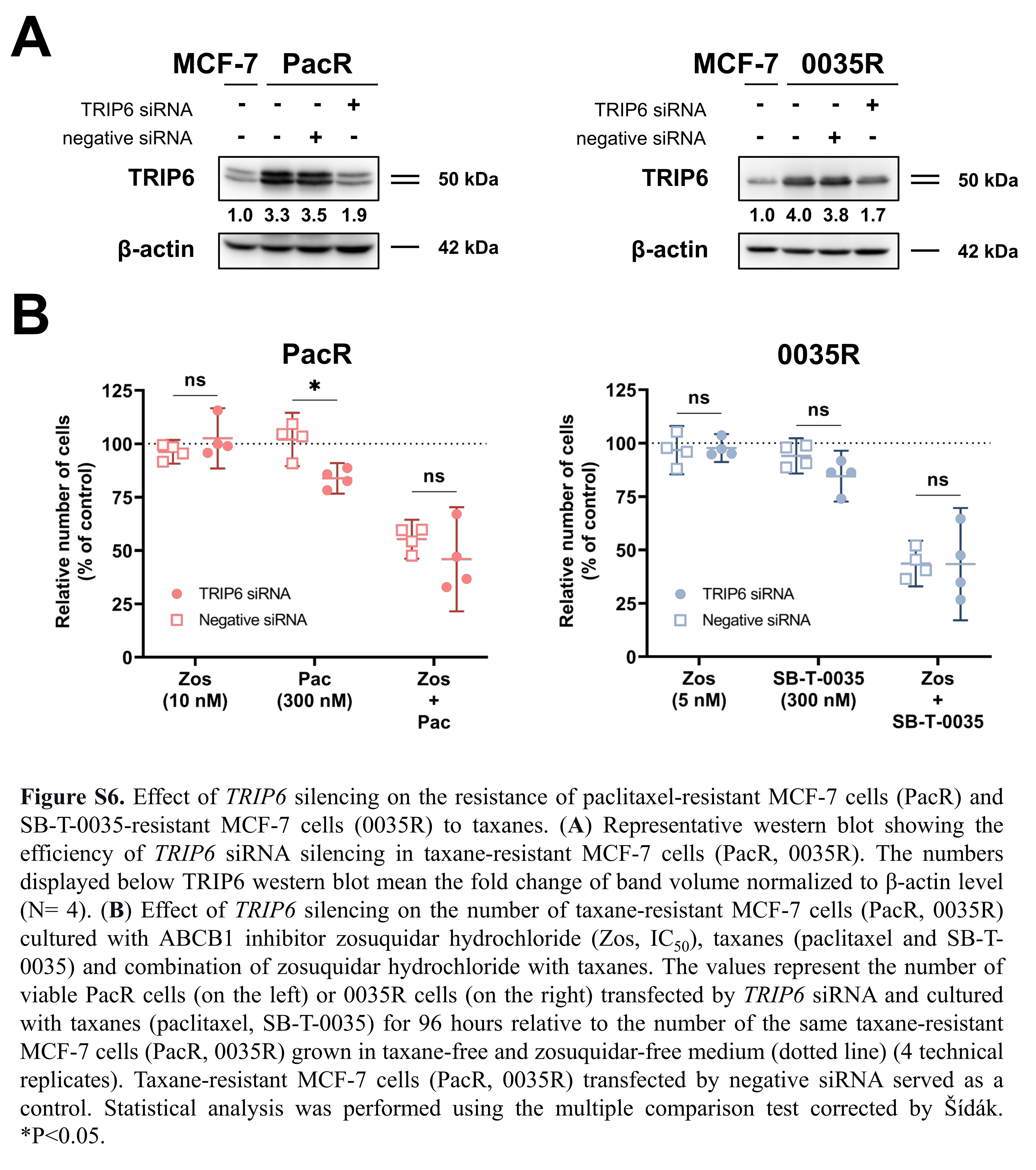

Supplement: Supplementary file 1 [file genes-14-00296-s001.zip › Supplementary Figure S6_Effect of TRIP6 silencing.tif]
